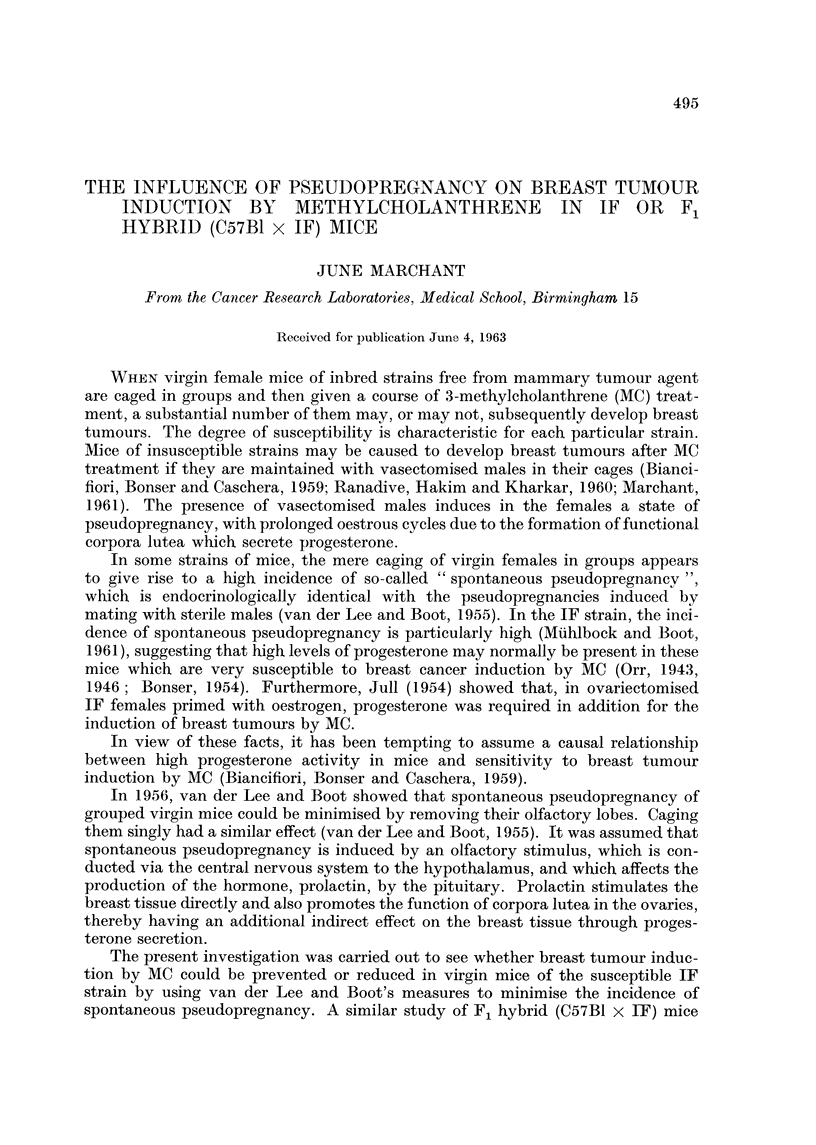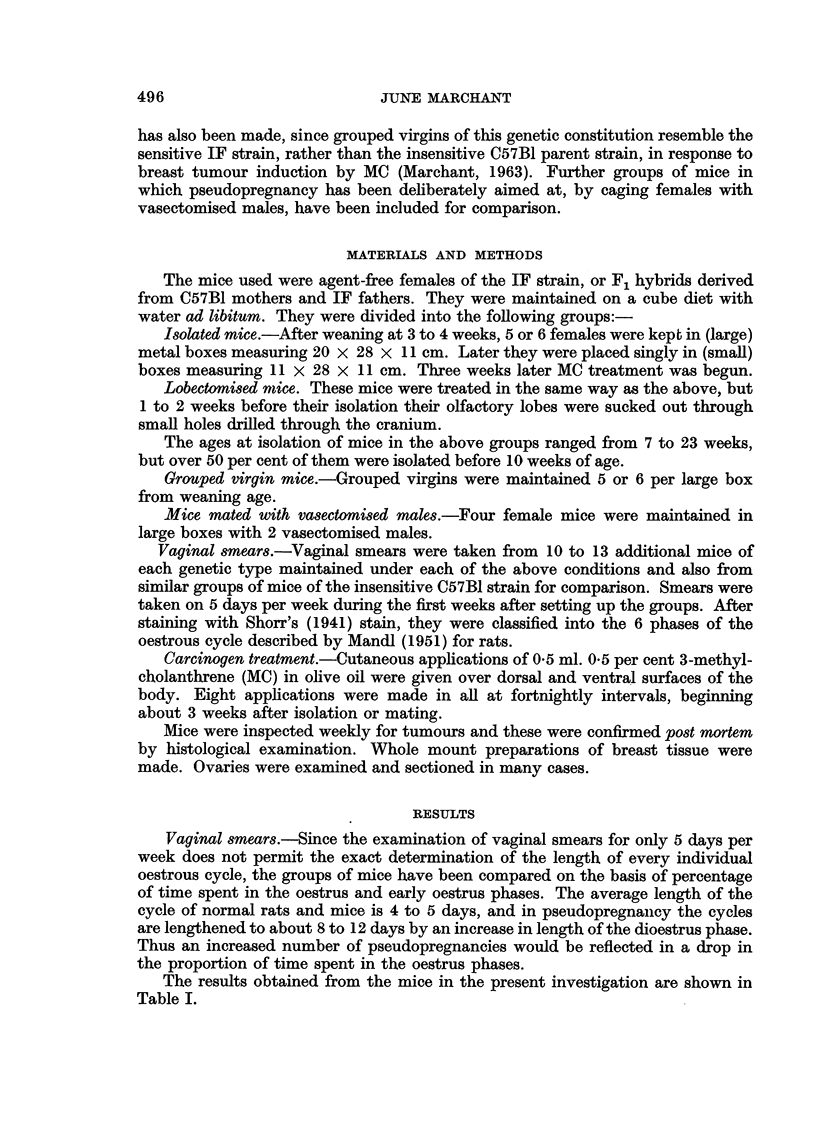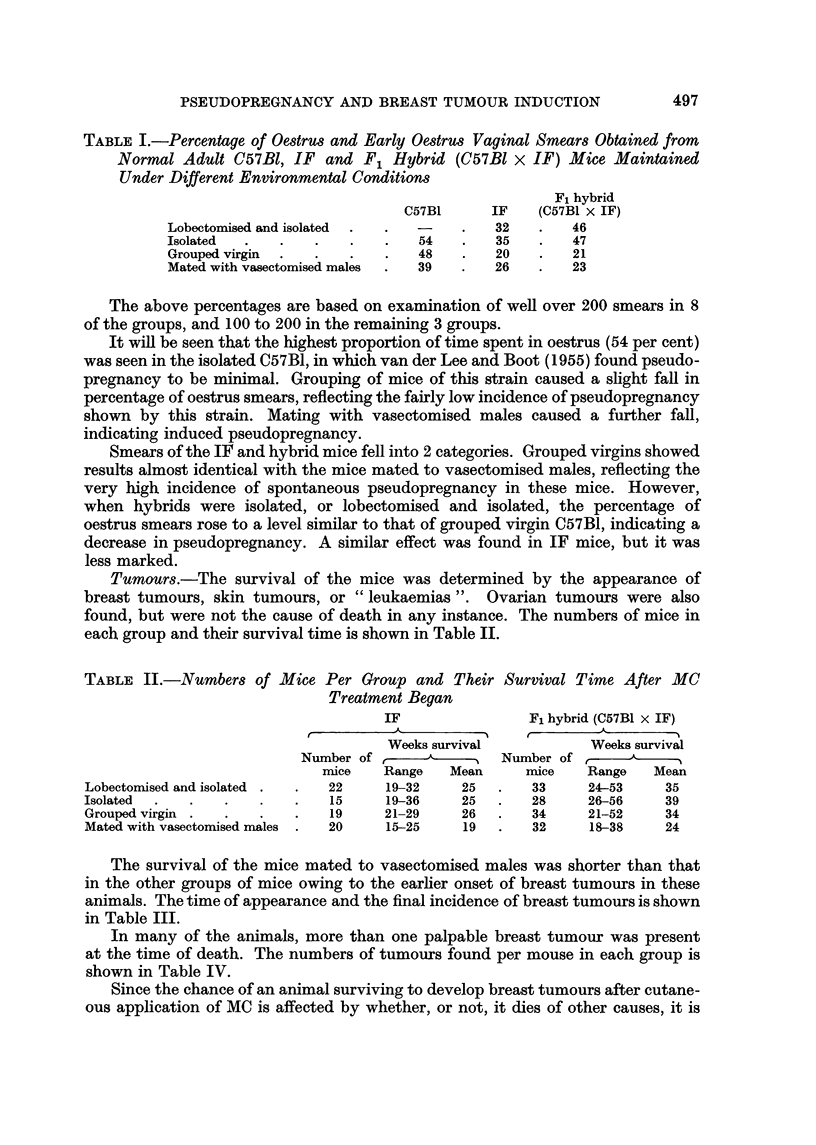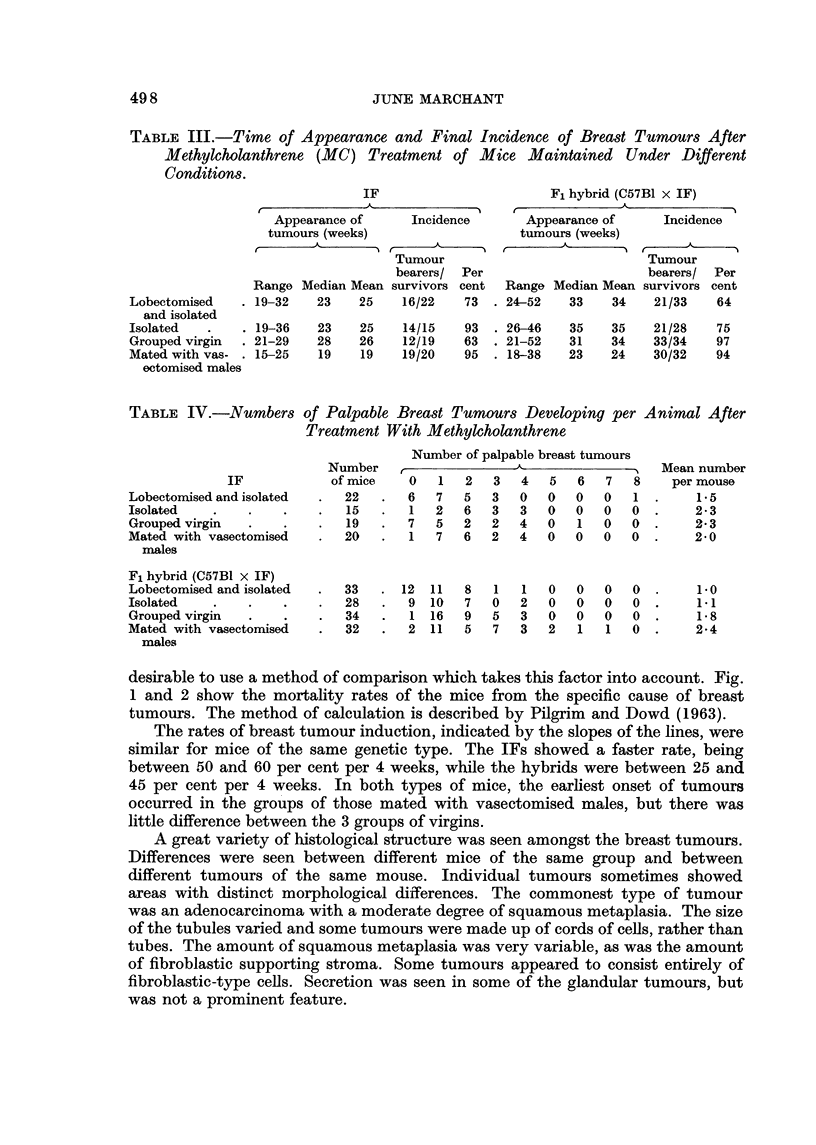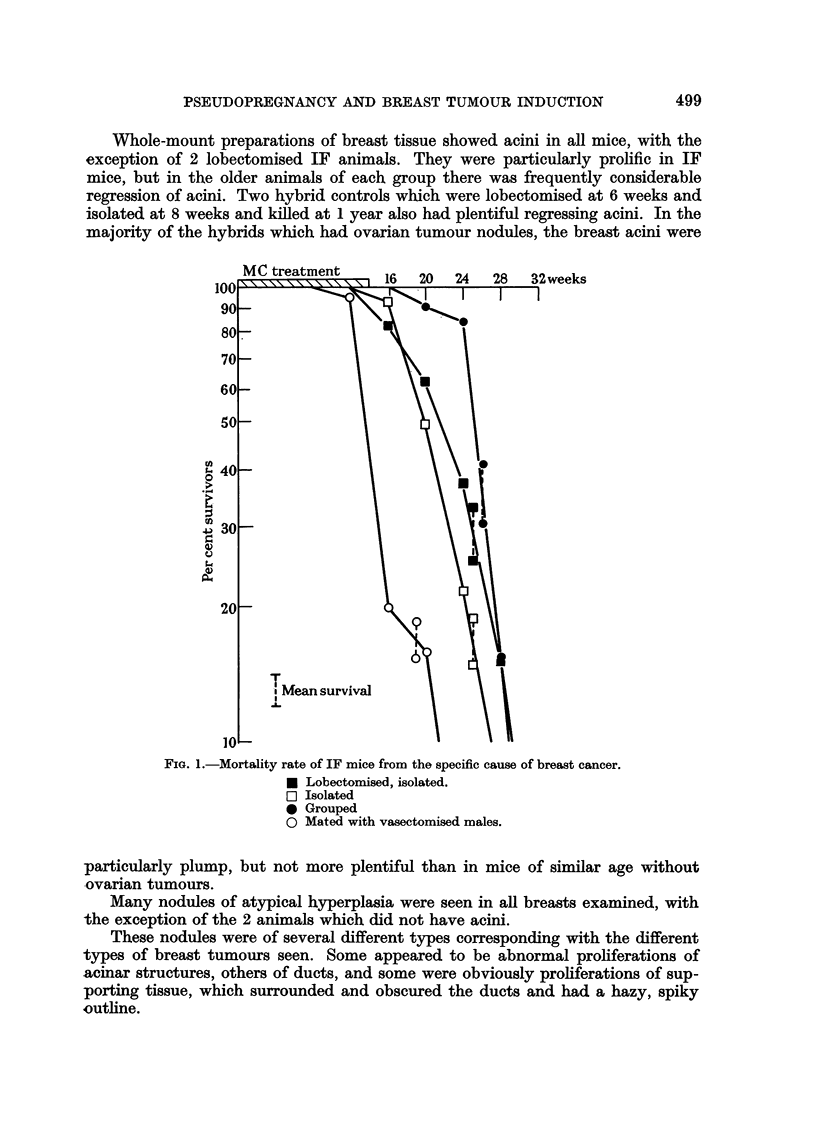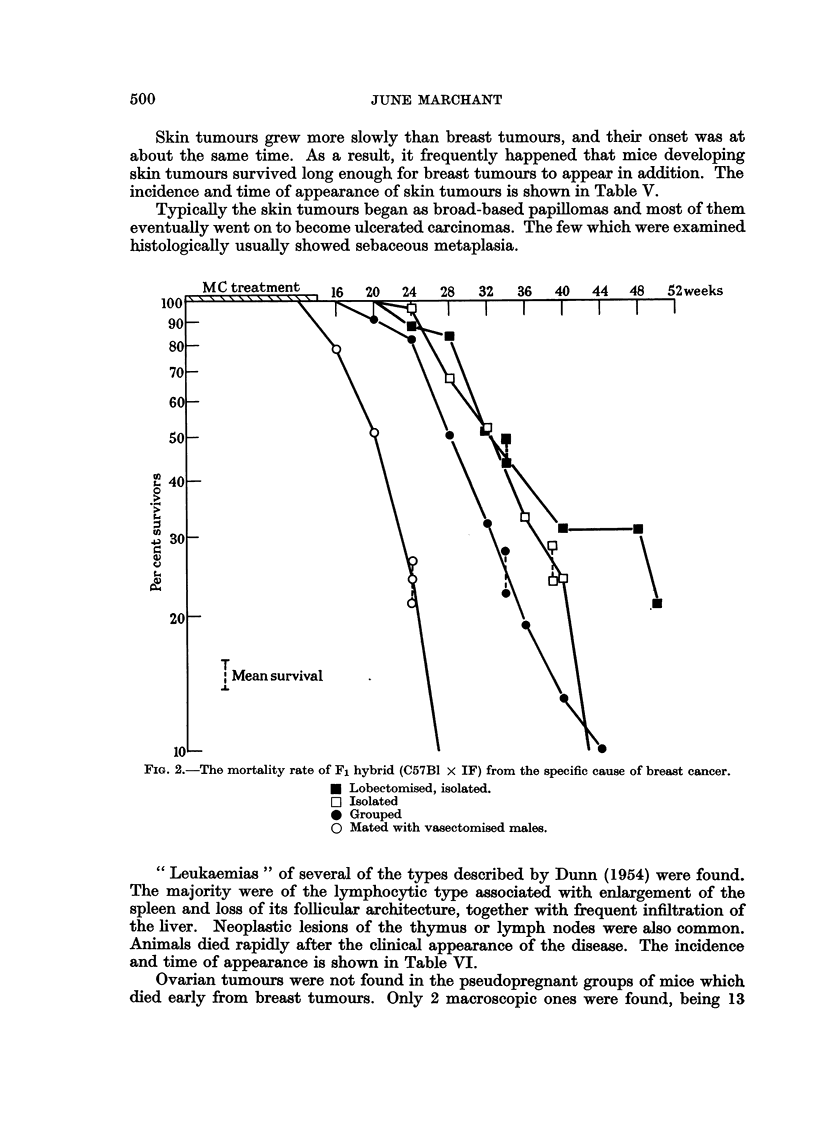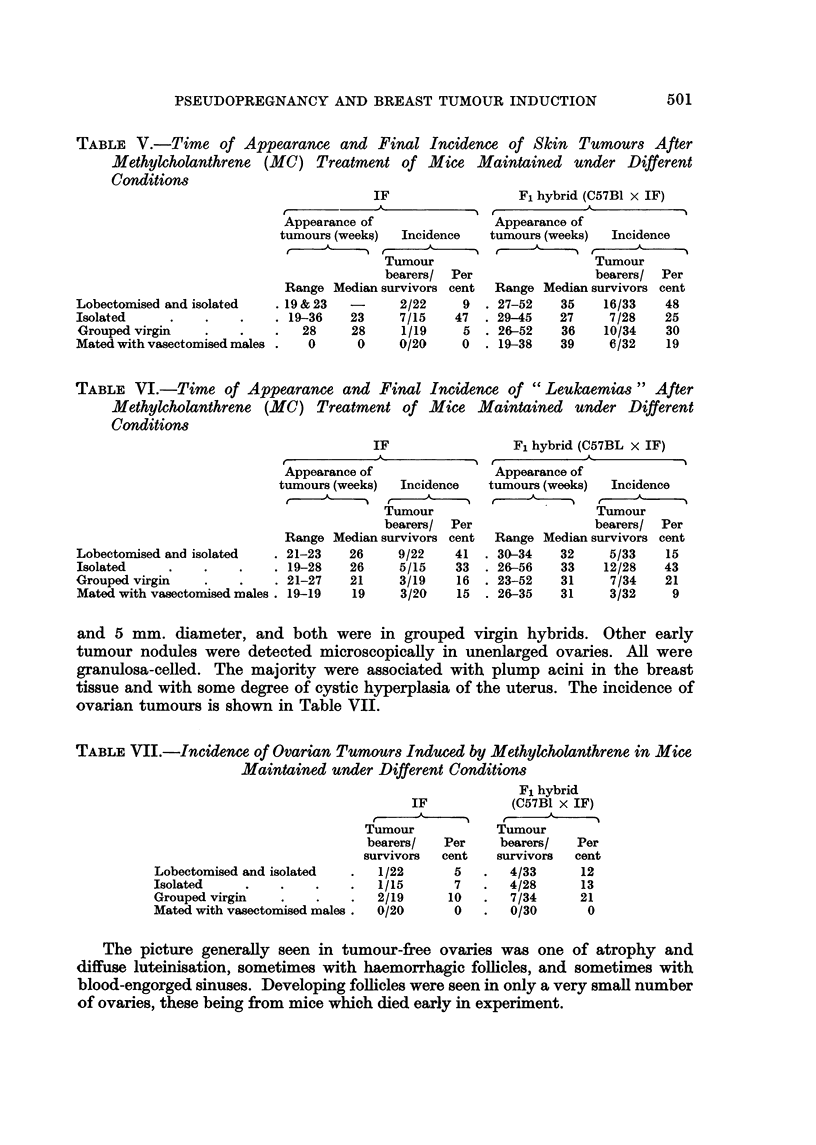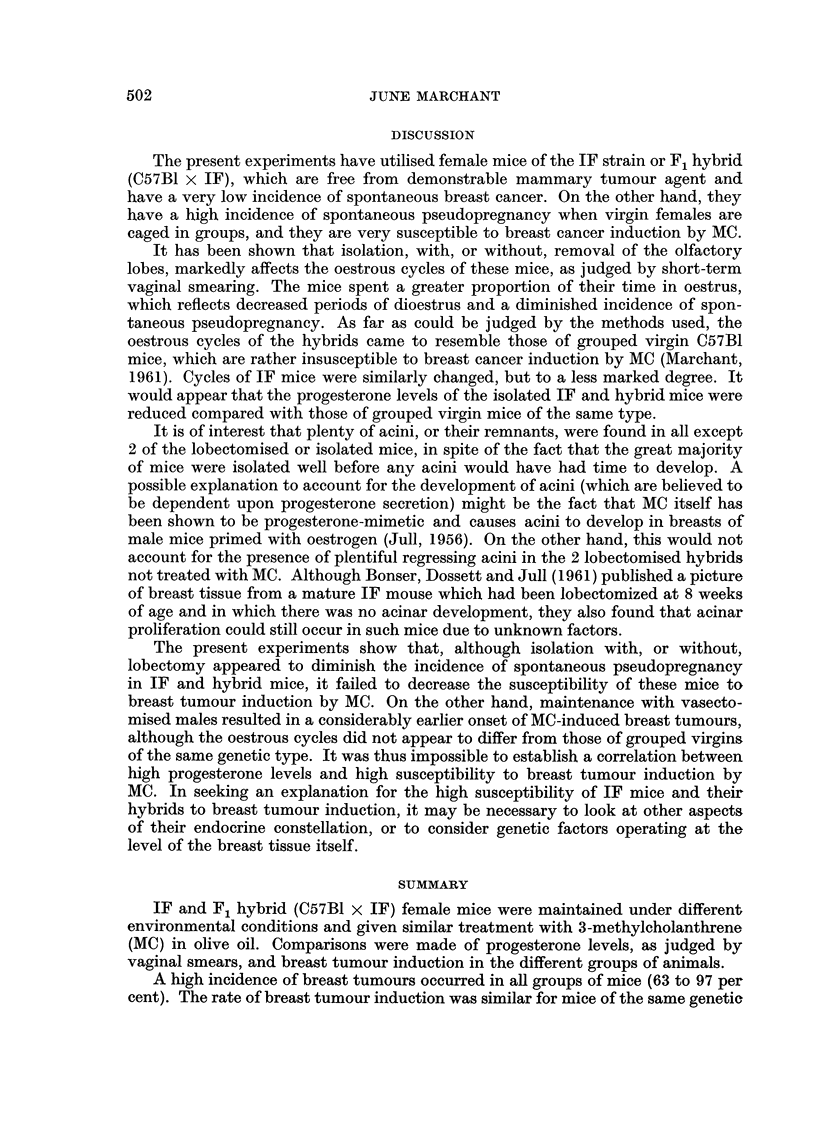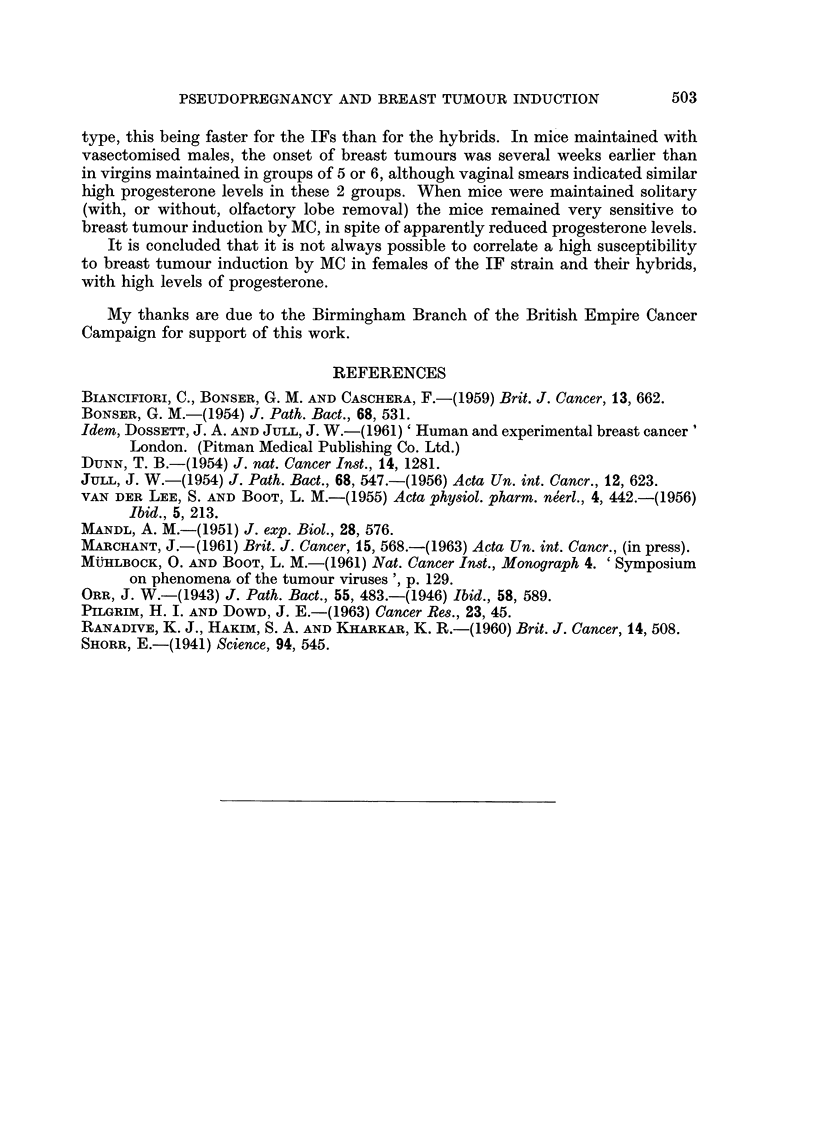# The Influence of Pseudopregnancy on Breast Tumour Induction by Methylcholanthrene in IF or F_1_ Hybrid (C57Bl × IF) Mice

**DOI:** 10.1038/bjc.1963.67

**Published:** 1963-09

**Authors:** June Marchant


### THE INFLUENCE OF PSEUDOPREGNANCY ON BREAST TUMOUR INDUCTION BY METHYLCHOLANTHRENE IN IF OR $F_1$ HYBRID (C57Bl × IF) MICE

JUNE MARCHANT

*From the Cancer Research Laboratories, Medical School, Birmingham* 15

Received for publication June 4, 1963

WHEN virgin female mice of inbred strains free from mammary tumour agent are caged in groups and then given a course of 3-methylcholanthrene (MC) treatment, a substantial number of them may, or may not, subsequently develop breast tumours. The degree of susceptibility is characteristic for each particular strain. Mice of insusceptible strains may be caused to develop breast tumours after MC treatment if they are maintained with vasectomised males in their cages (Biancifiori, Bonser and Caschera, 1959; Ranadive, Hakim and Kharkar, 1960; Marchant, 1961). The presence of vasectomised males induces in the females a state of pseudopregnancy, with prolonged oestrous cycles due to the formation of functional corpora lutea which secrete progesterone.

In some strains of mice, the mere caging of virgin females in groups appears to give rise to a high incidence of so-called " spontaneous pseudopregnancy ", which is endocrinologically identical with the pseudopregnancies induced by mating with sterile males (van der Lee and Boot, 1955). In the IF strain, the incidence of spontaneous pseudopregnancy is particularly high (Mühlbock and Boot, 1961), suggesting that high levels of progesterone may normally be present in these mice which are very susceptible to breast cancer induction by MC (Orr, 1943, 1946 ; Bonser, 1954). Furthermore, Jull (1954) showed that, in ovariectomised IF females primed with oestrogen, progesterone was required in addition for the induction of breast tumours by MC.

In view of these facts, it has been tempting to assume a causal relationship between high progesterone activity in mice and sensitivity to breast tumour induction by MC (Biancifiori, Bonser and Caschera, 1959).

In 1956, van der Lee and Boot showed that spontaneous pseudopregnancy of grouped virgin mice could be minimised by removing their olfactory lobes. Caging them singly had a similar effect (van der Lee and Boot, 1955). It was assumed that spontaneous pseudopregnancy is induced by an olfactory stimulus, which is conducted via the central nervous system to the hypothalamus, and which affects the production of the hormone, prolactin, by the pituitary. Prolactin stimulates the breast tissue directly and also promotes the function of corpora lutea in the ovaries, thereby having an additional indirect effect on the breast tissue through progesterone secretion.

The present investigation was carried out to see whether breast tumour induction by MC could be prevented or reduced in virgin mice of the susceptible IF strain by using van der Lee and Boot's measures to minimise the incidence of spontaneous pseudopregnancy. A similar study of $F_1$ hybrid (C57Bl × IF) mice has also been made, since grouped virgins of this genetic constitution resemble the sensitive IF strain, rather than the insensitive C57Bl parent strain, in response to breast tumour induction by MC (Marchant, 1963). Further groups of mice in which pseudopregnancy has been deliberately aimed at, by caging females with vasectomised males, have been included for comparison.

### MATERIALS AND METHODS

The mice used were agent-free females of the IF strain, or $F_1$ hybrids derived from C57Bl mothers and IF fathers. They were maintained on a cube diet with water *ad libitum*. They were divided into the following groups:—

*Isolated mice.*—After weaning at 3 to 4 weeks, 5 or 6 females were kept in (large) metal boxes measuring 20 × 28 × 11 cm. Later they were placed singly in (small) boxes measuring 11 × 28 × 11 cm. Three weeks later MC treatment was begun.

*Lobectomised mice.* These mice were treated in the same way as the above, but 1 to 2 weeks before their isolation their olfactory lobes were sucked out through small holes drilled through the cranium.

The ages at isolation of mice in the above groups ranged from 7 to 23 weeks, but over 50 per cent of them were isolated before 10 weeks of age.

*Grouped virgin mice.*—Grouped virgins were maintained 5 or 6 per large box from weaning age.

*Mice mated with vasectomised males.*—Four female mice were maintained in large boxes with 2 vasectomised males.

*Vaginal smears.*—Vaginal smears were taken from 10 to 13 additional mice of each genetic type maintained under each of the above conditions and also from similar groups of mice of the insensitive C57Bl strain for comparison. Smears were taken on 5 days per week during the first weeks after setting up the groups. After staining with Shorr's (1941) stain, they were classified into the 6 phases of the oestrous cycle described by Mandl (1951) for rats.

*Carcinogen treatment.*—Cutaneous applications of 0·5 ml. 0·5 per cent 3-methylcholanthrene (MC) in olive oil were given over dorsal and ventral surfaces of the body. Eight applications were made in all at fortnightly intervals, beginning about 3 weeks after isolation or mating.

Mice were inspected weekly for tumours and these were confirmed *post mortem* by histological examination. Whole mount preparations of breast tissue were made. Ovaries were examined and sectioned in many cases.

### RESULTS

*Vaginal smears.*—Since the examination of vaginal smears for only 5 days per week does not permit the exact determination of the length of every individual oestrous cycle, the groups of mice have been compared on the basis of percentage of time spent in the oestrus and early oestrus phases. The average length of the cycle of normal rats and mice is 4 to 5 days, and in pseudopregnancy the cycles are lengthened to about 8 to 12 days by an increase in length of the dioestrus phase. Thus an increased number of pseudopregnancies would be reflected in a drop in the proportion of time spent in the oestrus phases.

The results obtained from the mice in the present investigation are shown in Table I.

TABLE I.—*Percentage of Oestrus and Early Oestrus Vaginal Smears Obtained from Normal Adult C57Bl, IF and $F_1$ Hybrid (C57Bl × IF) Mice Maintained Under Different Environmental Conditions*

| | C57Bl | IF | $F_1$ hybrid (C57Bl × IF) |
|---|---|---|---|
| Lobectomised and isolated | — | 32 | 46 |
| Isolated | 54 | 35 | 47 |
| Grouped virgin | 48 | 20 | 21 |
| Mated with vasectomised males | 39 | 26 | 23 |

The above percentages are based on examination of well over 200 smears in 8 of the groups, and 100 to 200 in the remaining 3 groups.

It will be seen that the highest proportion of time spent in oestrus (54 per cent) was seen in the isolated C57Bl, in which van der Lee and Boot (1955) found pseudopregnancy to be minimal. Grouping of mice of this strain caused a slight fall in percentage of oestrus smears, reflecting the fairly low incidence of pseudopregnancy shown by this strain. Mating with vasectomised males caused a further fall, indicating induced pseudopregnancy.

Smears of the IF and hybrid mice fell into 2 categories. Grouped virgins showed results almost identical with the mice mated to vasectomised males, reflecting the very high incidence of spontaneous pseudopregnancy in these mice. However, when hybrids were isolated, or lobectomised and isolated, the percentage of oestrus smears rose to a level similar to that of grouped virgin C57Bl, indicating a decrease in pseudopregnancy. A similar effect was found in IF mice, but it was less marked.

*Tumours*.—The survival of the mice was determined by the appearance of breast tumours, skin tumours, or " leukaemias ". Ovarian tumours were also found, but were not the cause of death in any instance. The numbers of mice in each group and their survival time is shown in Table II.

TABLE II.—*Numbers of Mice Per Group and Their Survival Time After MC Treatment Began*

| | IF | | | $F_1$ hybrid (C57Bl × IF) | | |
|---|---|---|---|---|---|---|
| | | Weeks survival | | | Weeks survival | |
| | Number of mice | Range | Mean | Number of mice | Range | Mean |
| Lobectomised and isolated | 22 | 19–32 | 25 | 33 | 24–53 | 35 |
| Isolated | 15 | 19–36 | 25 | 28 | 26–56 | 39 |
| Grouped virgin | 19 | 21–29 | 26 | 34 | 21–52 | 34 |
| Mated with vasectomised males | 20 | 15–25 | 19 | 32 | 18–38 | 24 |

The survival of the mice mated to vasectomised males was shorter than that in the other groups of mice owing to the earlier onset of breast tumours in these animals. The time of appearance and the final incidence of breast tumours is shown in Table III.

In many of the animals, more than one palpable breast tumour was present at the time of death. The numbers of tumours found per mouse in each group is shown in Table IV.

Since the chance of an animal surviving to develop breast tumours after cutaneous application of MC is affected by whether, or not, it dies of other causes, it is

TABLE III.—*Time of Appearance and Final Incidence of Breast Tumours After Methylcholanthrene (MC) Treatment of Mice Maintained Under Different Conditions.*

| | IF | | | | | $F_1$ hybrid (C57Bl × IF) | | | | |
|---|---|---|---|---|---|---|---|---|---|---|
| | Appearance of tumours (weeks) | | | Incidence | | Appearance of tumours (weeks) | | | Incidence | |
| | Range | Median | Mean | Tumour bearers/ survivors | Per cent | Range | Median | Mean | Tumour bearers/ survivors | Per cent |
| Lobectomised and isolated | 19–32 | 23 | 25 | 16/22 | 73 | 24–52 | 33 | 34 | 21/33 | 64 |
| Isolated | 19–36 | 23 | 25 | 14/15 | 93 | 26–46 | 35 | 35 | 21/28 | 75 |
| Grouped virgin | 21–29 | 28 | 26 | 12/19 | 63 | 21–52 | 31 | 34 | 33/34 | 97 |
| Mated with vasectomised males | 15–25 | 19 | 19 | 19/20 | 95 | 18–38 | 23 | 24 | 30/32 | 94 |

TABLE IV.—*Numbers of Palpable Breast Tumours Developing per Animal After Treatment With Methylcholanthrene*

| IF | Number of mice | Number of palpable breast tumours 0 | 1 | 2 | 3 | 4 | 5 | 6 | 7 | 8 | Mean number per mouse |
|---|---|---|---|---|---|---|---|---|---|---|---|
| Lobectomised and isolated | 22 | 6 | 7 | 5 | 3 | 0 | 0 | 0 | 0 | 1 | 1·5 |
| Isolated | 15 | 1 | 2 | 6 | 3 | 3 | 0 | 0 | 0 | 0 | 2·3 |
| Grouped virgin | 19 | 7 | 5 | 2 | 2 | 4 | 0 | 1 | 0 | 0 | 2·3 |
| Mated with vasectomised males | 20 | 1 | 7 | 6 | 2 | 4 | 0 | 0 | 0 | 0 | 2·0 |
| $F_1$ hybrid (C57Bl × IF) | | | | | | | | | | | |
| Lobectomised and isolated | 33 | 12 | 11 | 8 | 1 | 1 | 0 | 0 | 0 | 0 | 1·0 |
| Isolated | 28 | 9 | 10 | 7 | 0 | 2 | 0 | 0 | 0 | 0 | 1·1 |
| Grouped virgin | 34 | 1 | 16 | 9 | 5 | 3 | 0 | 0 | 0 | 0 | 1·8 |
| Mated with vasectomised males | 32 | 2 | 11 | 5 | 7 | 3 | 2 | 1 | 1 | 0 | 2·4 |

desirable to use a method of comparison which takes this factor into account. Fig. 1 and 2 show the mortality rates of the mice from the specific cause of breast tumours. The method of calculation is described by Pilgrim and Dowd (1963).

The rates of breast tumour induction, indicated by the slopes of the lines, were similar for mice of the same genetic type. The IFs showed a faster rate, being between 50 and 60 per cent per 4 weeks, while the hybrids were between 25 and 45 per cent per 4 weeks. In both types of mice, the earliest onset of tumours occurred in the groups of those mated with vasectomised males, but there was little difference between the 3 groups of virgins.

A great variety of histological structure was seen amongst the breast tumours. Differences were seen between different mice of the same group and between different tumours of the same mouse. Individual tumours sometimes showed areas with distinct morphological differences. The commonest type of tumour was an adenocarcinoma with a moderate degree of squamous metaplasia. The size of the tubules varied and some tumours were made up of cords of cells, rather than tubes. The amount of squamous metaplasia was very variable, as was the amount of fibroblastic supporting stroma. Some tumours appeared to consist entirely of fibroblastic-type cells. Secretion was seen in some of the glandular tumours, but was not a prominent feature.

Whole-mount preparations of breast tissue showed acini in all mice, with the exception of 2 lobectomised IF animals. They were particularly prolific in IF mice, but in the older animals of each group there was frequently considerable regression of acini. Two hybrid controls which were lobectomised at 6 weeks and isolated at 8 weeks and killed at 1 year also had plentiful regressing acini. In the majority of the hybrids which had ovarian tumour nodules, the breast acini were

FIG. 1.—Mortality rate of IF mice from the specific cause of breast cancer.
■ Lobectomised, isolated.
□ Isolated
● Grouped
○ Mated with vasectomised males.

particularly plump, but not more plentiful than in mice of similar age without ovarian tumours.

Many nodules of atypical hyperplasia were seen in all breasts examined, with the exception of the 2 animals which did not have acini.

These nodules were of several different types corresponding with the different types of breast tumours seen. Some appeared to be abnormal proliferations of acinar structures, others of ducts, and some were obviously proliferations of supporting tissue, which surrounded and obscured the ducts and had a hazy, spiky outline.

Skin tumours grew more slowly than breast tumours, and their onset was at about the same time. As a result, it frequently happened that mice developing skin tumours survived long enough for breast tumours to appear in addition. The incidence and time of appearance of skin tumours is shown in Table V.

Typically the skin tumours began as broad-based papillomas and most of them eventually went on to become ulcerated carcinomas. The few which were examined histologically usually showed sebaceous metaplasia.

FIG. 2.—The mortality rate of $F_1$ hybrid (C57Bl × IF) from the specific cause of breast cancer.

■ Lobectomised, isolated.

□ Isolated

● Grouped

○ Mated with vasectomised males.

" Leukaemias " of several of the types described by Dunn (1954) were found. The majority were of the lymphocytic type associated with enlargement of the spleen and loss of its follicular architecture, together with frequent infiltration of the liver. Neoplastic lesions of the thymus or lymph nodes were also common. Animals died rapidly after the clinical appearance of the disease. The incidence and time of appearance is shown in Table VI.

Ovarian tumours were not found in the pseudopregnant groups of mice which died early from breast tumours. Only 2 macroscopic ones were found, being 13

TABLE V.—*Time of Appearance and Final Incidence of Skin Tumours After Methylcholanthrene (MC) Treatment of Mice Maintained under Different Conditions*

| | IF | | | | F1 hybrid (C57Bl × IF) | | | |
|---|---|---|---|---|---|---|---|---|
| | Appearance of tumours (weeks) | | Incidence | | Appearance of tumours (weeks) | | Incidence | |
| | Range | Median | Tumour bearers/ survivors | Per cent | Range | Median | Tumour bearers/ survivors | Per cent |
| Lobectomised and isolated | 19 & 23 | — | 2/22 | 9 | 27–52 | 35 | 16/33 | 48 |
| Isolated | 19–36 | 23 | 7/15 | 47 | 29–45 | 27 | 7/28 | 25 |
| Grouped virgin | 28 | 28 | 1/19 | 5 | 26–52 | 36 | 10/34 | 30 |
| Mated with vasectomised males | 0 | 0 | 0/20 | 0 | 19–38 | 39 | 6/32 | 19 |

TABLE VI.—*Time of Appearance and Final Incidence of "Leukaemias" After Methylcholanthrene (MC) Treatment of Mice Maintained under Different Conditions*

| | IF | | | | F1 hybrid (C57BL × IF) | | | |
|---|---|---|---|---|---|---|---|---|
| | Appearance of tumours (weeks) | | Incidence | | Appearance of tumours (weeks) | | Incidence | |
| | Range | Median | Tumour bearers/ survivors | Per cent | Range | Median | Tumour bearers/ survivors | Per cent |
| Lobectomised and isolated | 21–23 | 26 | 9/22 | 41 | 30–34 | 32 | 5/33 | 15 |
| Isolated | 19–28 | 26 | 5/15 | 33 | 26–56 | 33 | 12/28 | 43 |
| Grouped virgin | 21–27 | 21 | 3/19 | 16 | 23–52 | 31 | 7/34 | 21 |
| Mated with vasectomised males | 19–19 | 19 | 3/20 | 15 | 26–35 | 31 | 3/32 | 9 |

and 5 mm. diameter, and both were in grouped virgin hybrids. Other early tumour nodules were detected microscopically in unenlarged ovaries. All were granulosa-celled. The majority were associated with plump acini in the breast tissue and with some degree of cystic hyperplasia of the uterus. The incidence of ovarian tumours is shown in Table VII.

TABLE VII.—*Incidence of Ovarian Tumours Induced by Methylcholanthrene in Mice Maintained under Different Conditions*

| | IF | | F1 hybrid (C57Bl × IF) | |
|---|---|---|---|---|
| | Tumour bearers/ survivors | Per cent | Tumour bearers/ survivors | Per cent |
| Lobectomised and isolated | 1/22 | 5 | 4/33 | 12 |
| Isolated | 1/15 | 7 | 4/28 | 13 |
| Grouped virgin | 2/19 | 10 | 7/34 | 21 |
| Mated with vasectomised males | 0/20 | 0 | 0/30 | 0 |

The picture generally seen in tumour-free ovaries was one of atrophy and diffuse luteinisation, sometimes with haemorrhagic follicles, and sometimes with blood-engorged sinuses. Developing follicles were seen in only a very small number of ovaries, these being from mice which died early in experiment.

### DISCUSSION

The present experiments have utilised female mice of the IF strain or $F_1$ hybrid (C57Bl × IF), which are free from demonstrable mammary tumour agent and have a very low incidence of spontaneous breast cancer. On the other hand, they have a high incidence of spontaneous pseudopregnancy when virgin females are caged in groups, and they are very susceptible to breast cancer induction by MC.

It has been shown that isolation, with, or without, removal of the olfactory lobes, markedly affects the oestrous cycles of these mice, as judged by short-term vaginal smearing. The mice spent a greater proportion of their time in oestrus, which reflects decreased periods of dioestrus and a diminished incidence of spontaneous pseudopregnancy. As far as could be judged by the methods used, the oestrous cycles of the hybrids came to resemble those of grouped virgin C57Bl mice, which are rather insusceptible to breast cancer induction by MC (Marchant, 1961). Cycles of IF mice were similarly changed, but to a less marked degree. It would appear that the progesterone levels of the isolated IF and hybrid mice were reduced compared with those of grouped virgin mice of the same type.

It is of interest that plenty of acini, or their remnants, were found in all except 2 of the lobectomised or isolated mice, in spite of the fact that the great majority of mice were isolated well before any acini would have had time to develop. A possible explanation to account for the development of acini (which are believed to be dependent upon progesterone secretion) might be the fact that MC itself has been shown to be progesterone-mimetic and causes acini to develop in breasts of male mice primed with oestrogen (Jull, 1956). On the other hand, this would not account for the presence of plentiful regressing acini in the 2 lobectomised hybrids not treated with MC. Although Bonser, Dossett and Jull (1961) published a picture of breast tissue from a mature IF mouse which had been lobectomized at 8 weeks of age and in which there was no acinar development, they also found that acinar proliferation could still occur in such mice due to unknown factors.

The present experiments show that, although isolation with, or without, lobectomy appeared to diminish the incidence of spontaneous pseudopregnancy in IF and hybrid mice, it failed to decrease the susceptibility of these mice to breast tumour induction by MC. On the other hand, maintenance with vasectomised males resulted in a considerably earlier onset of MC-induced breast tumours, although the oestrous cycles did not appear to differ from those of grouped virgins of the same genetic type. It was thus impossible to establish a correlation between high progesterone levels and high susceptibility to breast tumour induction by MC. In seeking an explanation for the high susceptibility of IF mice and their hybrids to breast tumour induction, it may be necessary to look at other aspects of their endocrine constellation, or to consider genetic factors operating at the level of the breast tissue itself.

### SUMMARY

IF and $F_1$ hybrid (C57Bl × IF) female mice were maintained under different environmental conditions and given similar treatment with 3-methylcholanthrene (MC) in olive oil. Comparisons were made of progesterone levels, as judged by vaginal smears, and breast tumour induction in the different groups of animals.

A high incidence of breast tumours occurred in all groups of mice (63 to 97 per cent). The rate of breast tumour induction was similar for mice of the same genetic type, this being faster for the IFs than for the hybrids. In mice maintained with vasectomised males, the onset of breast tumours was several weeks earlier than in virgins maintained in groups of 5 or 6, although vaginal smears indicated similar high progesterone levels in these 2 groups. When mice were maintained solitary (with, or without, olfactory lobe removal) the mice remained very sensitive to breast tumour induction by MC, in spite of apparently reduced progesterone levels.

It is concluded that it is not always possible to correlate a high susceptibility to breast tumour induction by MC in females of the IF strain and their hybrids, with high levels of progesterone.

My thanks are due to the Birmingham Branch of the British Empire Cancer Campaign for support of this work.

### REFERENCES

Biancifiori, C., Bonser, G. M. and Caschera, F.—(1959) *Brit. J. Cancer*, **13**, 662.
Bonser, G. M.—(1954) *J. Path. Bact.*, **68**, 531.
*Idem*, Dossett, J. A. and Jull, J. W.—(1961) ' Human and experimental breast cancer ' London. (Pitman Medical Publishing Co. Ltd.)
Dunn, T. B.—(1954) *J. nat. Cancer Inst.*, **14**, 1281.
Jull, J. W.—(1954) *J. Path. Bact.*, **68**, 547.—(1956) *Acta Un. int. Cancr.*, **12**, 623.
van der Lee, S. and Boot, L. M.—(1955) *Acta physiol. pharm. néerl.*, **4**, 442.—(1956) *Ibid.*, **5**, 213.
Mandl, A. M.—(1951) *J. exp. Biol.*, **28**, 576.
Marchant, J.—(1961) *Brit. J. Cancer*, **15**, 568.—(1963) *Acta Un. int. Cancr.*, (in press).
Mühlbock, O. and Boot, L. M.—(1961) *Nat. Cancer Inst., Monograph* **4**. ' Symposium on phenomena of the tumour viruses ', p. 129.
Orr, J. W.—(1943) *J. Path. Bact.*, **55**, 483.—(1946) *Ibid.*, **58**, 589.
Pilgrim, H. I. and Dowd, J. E.—(1963) *Cancer Res.*, **23**, 45.
Ranadive, K. J., Hakim, S. A. and Kharkar, K. R.—(1960) *Brit. J. Cancer*, **14**, 508.
Shorr, E.—(1941) *Science*, **94**, 545.